# In conversation with…Thomas Frederic Hornbein

**DOI:** 10.1186/s13728-016-0053-1

**Published:** 2016-10-11

**Authors:** Ned Gilbert-Kawai

**Affiliations:** UCL Centre for Altitude, Space and Extreme Environment Medicine, Institute of Sport and Exercise Health, 170 Tottenham Court Road, London, W1T 7HA UK

## Abstract

Whilst attending a conference in the not too distant past, I was fortunate enough to be seated at lunch next to a very eminent octogenarian academic. Conversation ensued, and I was instantly captivated by his stories. These were not narratives filled with scientific facts, but rather anecdotes of his life and the pathways he had taken to get where he was today. Fascinated by such accounts, I set about the task of interviewing persons of scientific acclaim, to learn more about their life stories and unwritten tales. ‘In conversation with…’ therefore offers readers a chance to share an abridged version of these conversations. (In view of the need to condense 4 h of conversation, the original interview text has been abridged, and then reviewed, revised, and approved by TFH.)

“I’m game to talk a bit about Everest and the West Ridge, but it is a part of my life that’s been heavily mined. To have someone curious about the rest of my life, not least my life as doctor, teacher, and researcher, is a treat.”

## Q. You were born in St Louis in 1930. What did your parents do?

My mom was a classical housewife, raising kids. My dad was head of advertising at the major department store in St Louis. I remember as a kid visiting the 11th floor of this big store where there was a large network of offices. All these young people were doing drawings of women modelling clothes and such things for the newspaper ads. It was kind of magical, and Dad was obviously very caring and beloved by his staff.

## Q. Were your parents influential on your later life and choice of career?

One facet of the way my two sisters and I were brought up was that our parents really believed that, even when we were young, we had brains of our own and could think and figure things out for ourselves. It provided us with a sense that we were self-empowered, and I think that it was a real gift to us. I guess while there were obviously a lot of doubts growing up—that’s part of the essence of the game—I never had to struggle with a fragile self-image. I always felt challenged and willing to take risks; and yes, I guess that has directed my life in some important ways.

## Q. A lot of your life has been spent in the mountains, but were you a very outdoor-oriented child?

Well, growing up in St Louis I just wanted to be outside. In my book, *Everest the West Ridge*, the four prefaces that have come out over the last half-century pick up on my childhood —how I used to climb trees and houses when I was a little kid, just because there was some ape in me! It wasn’t, however, until I was 13 and my parents sent me to camp here in Colorado that I discovered mountains. And that really was the major pivotal event in my life that has led to everything else, including this conversation that we’re having right now.

## Q. Your first degree was as a geology major?

I went to the University of Colorado to major in geology, but devoted more time to climbing on the rocks than studying them. The University of Colorado has always had a reputation as a bit of a party school, and I had a lot of time to go out and scamper on the Flatirons. Meanwhile, I’d gotten very involved in mountain rescue and first aid, so after 3 years at university I changed my major, pieced together something of a mixed major in geology, mineralogy, and chemistry, and devoted my final year to doing what I had to do to get into medical school.

## Q. How did you find medical school?

Both my geology major and medical school times were great. When I advise young people now, I look on college as a time of discovering yourself. It’s the first time you’re away from your parents and family, and all the book-learning is a piece of it, but only a part. For me, those were very formative years, going out and climbing on the rocks, doing dumb stuff, teaching myself, and fortunately surviving! It laid the groundwork for precepts of commitment, problem-solving, and risk-taking—all of which are core ingredients of my life.

## Q. I read that when you were at medical school, you had dreams of being a general practitioner out in Colorado. What prompted the change of heart?

Washington University in St Louis was a very research-oriented medical school, and they quickly disavowed me of the idea of general practise. My professor of surgery at the time suggested I should think about anaesthesiology—pretty much a nonentity back then. Well, I had no exposure to it except for holding retractors during my surgical rotations and seeing the nurse-anaesthetists with the patients, and at first I was not terribly curious. However, it seemed like it might provide a way to take good care of patients and still pursue research in another part of my life without having the two interests competing with each other. So I thought, ‘Sure, why not?’

Back then, anaesthesia was on the cusp of transformation—from an art form if you will, with a few basic anaesthetics like ether, cyclopropane, and even chloroform. When I started my residency we didn’t even have an EKG monitor; it was a finger on the pulse and a blood pressure cuff. The development of blood gas measurement was really the first inroad into the transformation to what we know as anaesthesia today—a subject based on a profound and wonderful physiology and pharmacology. My generation was right in the middle of that whole process of transformation where the ability to do what we did in our physiology classes became a reality in the operating room. The physiology was so fascinating and compelling, and the evolution of anaesthesia that has taken place in my lifetime is pretty phenomenal to me.

## Q. When did your interest in research arise?

Almost as soon as I got to medical school, I started reading the literature on altitude and acclimatisation. In those days, it wasn’t a huge inventory, but not a small one either. One of the papers I came across was written by a Peruvian named Hugo Chiodi. It was seminal to my life, for it led to my first study, when I was in my senior year of medical school. We were given a 6-week elective where we could do whatever we wanted, so I concocted a rather simple-minded study to assess the link between haematocrit and ventilation, which involved pedalling on a bicycle. Well, as EPO didn’t exist back then, to get a high haematocrit we had to transfuse the subject with five units of blood. Perhaps unsurprisingly, the only subject who was willing to do that was me! Fortunately, the study got published in the *Journal of Applied Physiology*, which I suspect would not be the case nowadays for it had only one subject, hence no statistics; but it was pretty explicit as an anecdote.

I then became involved with Dr. Albert Roos, a little Dutch man whose parents sent him from Holland to the States just before Hitler moved in. His area of research interests at the time had to do with lung gas exchange, ventilation, and pulmonary blood flow, so when I came knocking on his door with my unrelated questions, whilst it wasn’t quite in his area of interest, he took me in and became my mentor. Interestingly, my questions must have got Albert thinking about how pH is regulated, for his whole research career shifted towards pursuing this subject and eventually he became the world’s leading authority on how the brain regulates intracellular pH. Sadly, I can’t take any credit for anything except being, coincidentally, the catalyst for stirring his terribly inquisitive mind to start thinking about something he hadn’t thought about before.

## Q. After your anaesthesia residency, you moved from Washington University in St Louis to Seattle, but I believe that was not an easy choice

I had finished my training with Albert and my anaesthesia residency, had been picked up by the Navy briefly, and was planning to go to Everest. However, before leaving for Everest, I had to make a decision: on my return would I go back to Washington University in St Louis, or to Seattle where I had interned and fallen in love with the Pacific North-West and its mountains? I was torn as to whether I had an obligation to go back to where they had trained me, and to some degree even subsidised me. Eventually I took advice on the matter from my brother-in-law—a neurologist at Washington University, named Billy Landau. He wrote me a letter that I still remember vividly: ‘Your obligation to the institution that trains you is to do the best you can wherever you go.’ i.e., go where your heart takes you, commit yourself, and do it well… and that’s how I ended up in Seattle for 43 years.

## Q. Aside from your clinical research, you also had a passion for the mountains that then led to research relating to oxygen masks…

In 1960, I was invited to come along as the climbing doctor for the America–Pakistan Karakoram Expedition to Masherbrum. This was somewhere between my anaesthesia residency and my fellowship years. There we planned to use the masks the Swiss had used on their successful ascent of Everest in ‘56. However, when we got up high and put them on, we found ourselves ripping the mask off and gasping for air. It got me thinking about the problems with the mask, one of which was obviously a pretty high resistance to breathing through the inspiratory and expiratory valves. So I thought, ‘Well, maybe one can come up with a better mask.’ And I came up with what I still think is a pretty damned creative idea for a mask.

Funnily enough, whilst I had developed the concept for it, I hadn’t figured out how I was actually going to make the mask. As it happened, one evening I gave a talk about Masherbrum to our medical staff at the Washington University Medical School, and one of the surgeons had brought a patient down to listen to my talk because he thought he would be interested. Now this guy, Fred Maytag, was 3 days post-op from a pelvic exenteration for colon cancer, and whilst he didn’t quite make it through the whole talk, he heard about half of it before he had to go and get horizontal again. So I got acquainted with Fred, and after I’d done my list each day, I’d go up and chat with him. Well, Fred listened to my thoughts on this mask idea, and it turned out that he was the head of a company that made washing machines… which then diversified somewhat and took on the job of taking my concept and turning it into a mask, which I designated as the Maytag mask.

## Q. Following your time on Masherbrum you were drafted for military service, though this ended slightly prematurely

Yes, after my residency and fellowship were done, I started my military service and I got assigned to the US Navy. By this time, I had already gotten an invitation from Norman Dyhrenfurth to go to Everest, so I asked the admiral at the Naval Hospital in San Diego where I was stationed for permission to join this expedition. Well, he was OK with the idea; however, the Bureau of Medicine and Surgery of the Navy in Washington wasn’t, and they turned me down not once, but twice. Then 1 day, out of the blue I got a call from some admiral in the Bureau who said ‘I’ve been instructed to let you go to Everest, but in order to go you’re going to have to be discharged from the Navy.’ Well, that was that, and I was honourably discharged and went off to Everest. About 30 years later, at an Everest IMAX film premier in Denver, I found out how that telephone call came about. Willi Unsoeld, whom I later climbed the West Ridge with, was in the newly formed Peace Corps, and his boss, Robert Sergeant Shriver, happened to be the brother-in-law of a guy named John F. Kennedy. So JFK kindly called his Secretary of Defense, Robert McNamara, and I was soon discharged and once again a member of the team.

## Q. Whilst I am utterly fascinated by your incredible achievement of summiting Everest via the West Ridge, I appreciate you have been interviewed about these exploits hundreds of times, and I imagine you are probably fed up of talking about them?

Yes, I’m game to talk a bit about Everest and the West Ridge, but it is a part of my life that’s been heavily mined. To have someone curious about the rest of my life, not least my life as doctor, teacher, and researcher is a treat.

## Q. The events of your successful summiting on the American Mount Everest Expedition of 1963 are encapsulated in your wonderful book *Everest: The West Ridge*. Did you know when you were out there, that you were going to write the book?

You know, that’s a good question. I don’t know when I started thinking about it. I knew James Ramsey Ullman was assigned to write the official account of the expedition; unfortunately however, he didn’t get to Base Camp as had been the plan. Now I had been reared and grown up on his writing—my bible as a teenager was *High Conquest*, his history of mountaineering. His tendency was to look on mountaineers as super-human beings and make it all very heroic. To me, however, the remarkable thing about our expedition wasn’t our climb so much, but rather the dynamics of one main team split into two smaller teams: the ‘South Col-ers’ and the ‘West Ridge-ers’; two groups intensely living together, competing on two different routes and not putting ice-axes in each other’s heads. It’s not often you get that dynamic of two parties, and there was a potential for intense friction along the way. We grumbled, we disagreed, we negotiated, we solved problems, and above all we made it all work. So to me, the story that really wanted to be told was not a super-human story, but a human story in an uncommon setting. And that’s what motivated me to write about it. But I can tell you that writing the book was a lot harder than climbing the mountain!

## Q. It was also very interesting to me to read through the four prefaces written over the last 50 years for each subsequent edition, and see how they have changed over time

I must say I agree with you on that. When the last edition came along I really struggled. It forced me to look back on a half-century of life, and try to put some pieces together that I hadn’t done. Early on, when I re-read the book, I would read it and think of all the things I should have said differently. But then, after about 10 years, that became irrelevant. Reading it now, it’s hard to believe that’s the same Tom Hornbein that I am now. I look at my body now, trying to go out and do the stuff I used to do with my younger friends, and it doesn’t behave like it used to. But, whilst adapting to older age is no small challenge, I look at what I can do and what I can revel in, and think how lucky I am. The fact is you just keep doing as long as you can if that’s your passion, and you accept that it’s done somewhat differently than it might have been in your younger years. For me this phase in life is really rich and I feel very lucky indeed.

## Q. On your return from Everest you moved to Seattle where you became an assistant professor of anaesthesiology…

Yes, I had interned there in 1956–1957. The guy who was running the program then was Lucien Morris, the inventor of the copper kettle vaporiser for ether. As an intern, I can remember watching his residents. They were required to keep these copper vaporisers and the copper table-tops to which they were connected for dissipation of cold polished to the hilt. Lucien was a somewhat-opinionated, but very bright man. I could see that we wouldn’t have gone well together. However, in the interim, when I was doing my residency, the medical school in Seattle recruited John Bonica to be the first Chair of Anaesthesia. He was one of the pioneers of regional anaesthesia in the US—a skill which he had taught himself during the Second World War on injured soldiers. Additionally, he was built like a tank, and had paid his way through his years in college and medical school by going out and wrestling farm boys! He was not a conventional academic, but he knew what he wanted in the way of a department, which was to be the greatest department in the world, so he began to recruit faculty with that in mind. After writing to him, I realised he would be examining me at my board exams. So I grabbed a lift over to Houston from San Diego in a Navy plane, jumped off and went to the exam, and then asked him for an interview. Well, we had dinner, and he just hired me on the spot. His biggest concern was whether I’d always be running off on expeditions or not! So that was the beginning of it all, and I set up my lab and continued studying peripheral chemoreceptor function. This was supported by a new form of National Institute of Health support called a Research Career Development Award. I could spend 80 % of my time in the laboratory and a day a week in the operating room.

## Q. And during those 43 years, a large amount of research followed

At the beginning of 1970, I switched my research focus from peripheral chemoreceptors to central chemoreceptors. This was undoubtedly influenced by John Severinghaus and another of his protégés, a Dane named Soren Sorensen. Soren came and worked in my lab for a while, and I did a sabbatical at the Rockefeller Institute in Copenhagen. I was also busy writing a chapter for a physiology textbook on the control of ventilation, and this got me thinking about how CSF pH is regulated. So I embarked on a whole series of studies relating to respiratory and metabolic acid-based derangements; however, I can’t say they have changed the Earth very much.

I then started getting involved in other stuff in the department, like teaching and administering the residency programme. Pretty soon however, I found myself with so many challenging and interesting things to do, that I just felt that I was doing a lousy job on everything. I probably wasn’t I guess, but by my own standards I felt I was. I can remember talking to Dr. Bonica, explaining how overloaded I felt. He said, ‘Write down on a piece of paper everything you’re doing, put it in order of what you like doing most on there, and then let’s look at it.’ So I did just that, and we knocked off stuff at the bottom and offered it to others to do—a valuable lesson learnt, which I have passed on to others.

My research really ended when I became department Chair in 1978. After that I saw my role, in terms of my science, to be to do everything I could to help those in my faculty who, like me, were really interested in research. I saw it as part of my job description to be able to critique, and help those people write papers, write grants, and get funding.

## Q. And teaching was also very important to you?

I loved to teach. Of course, you’re virtually never giving an anaesthetic by yourself in academic anaesthesia, you’re with a trainee, and that was probably the part of it that gave me the most pleasure. I felt that I needed to be an informed, active, and an exemplary clinical presence, and I needed to know what was going on because research and teaching are critical pieces of an academic department, and the foundation on which it’s based is patient care. The bottom line, and what I wanted for my residents to take away from their learning experience and out into practise, is taking good care of people and doing it exceedingly well. I’ve maintained contact with only a tiny fraction of all those we trained, and watching them doing what they are doing now makes one glow. When you think about immortality, it’s an anonymous one, but it’s watching these people impact the world around them and impact others’ lives as much as I was able to impact their lives. I just feel so proud of what some of them are accomplishing. It is beyond anything I could have ever imagined doing myself.

## Q. Did you continue your clinical commitments throughout this time?

The flavours of my chair life were administration, (but who would ever, in their right mind, want to be doing that?), my research, my interaction with young faculty to help counsel and guide them, and I would spend 1 day a week clinically in the operating room. We had four hospitals, so I would rotate monthly amongst these four sites. I would also take call at night, which many chairmen didn’t and don’t do, but I did that for two reasons: first of all I felt it was important to do this as a role model; second, there was so much I could observe and learn by being there. I could see what was going on in the operating rooms and what were the problems. As was the case on Everest where there were two teams, in the hospital there’s always a tension of sorts between surgeons, anaesthetists, and others. The operating room is very much like that expedition where you need to keep people working together and functioning together in good style. So as one of the department chairs, clinically I was a participant in trying to make that environment a really constructive one where you could repress the impulses of prima donnas; and it became a team endeavour between nursing, anaesthesia, and surgery. There was a lot of evolution to make it good, and it worked better in some places than in others, but that was all part of the challenge.

## Q. With such a busy professional life, how did you find retirement?

In 1998 I gave my last anaesthetic, and when I stepped out of the operating room, in a sense I shut the door on nearly a half-century of my life and moved on. I figured that the world doesn’t need old anaesthesiologists; they’ve got younger people like you. I came to realise that you either stop before you need to, or after you should have. You can apply that to many other things in life, but in this case it really gets down to the nub of what is in the best interests of patients, not what’s in the best interests of the passionate doc. And one of the things I have occasionally found myself doing with colleagues of my generation is counselling them through that transition. For some it’s easy, but for others anaesthesia has been their life and thinking of stopping is almost unimaginable. But it isn’t. Life goes on, and once they’ve done it they discover exactly that. I never had a sense that I was going to be bored, and that there wouldn’t be things to challenge a still-curious mind; it was more a matter, once again, of trying to figure out which ones to select.

I had been very much involved in the future direction of the Association of University Anaesthesiologists. It’s a vital organisation which enabled one of my more creative moments during my chair life. This had to do with addiction, which was a topic that I didn’t know I was going to have so much involvement with, but is a very real problem in our specialty. One night, in the early 90s, I was on call and around midnight finished in the operating rooms and went up to my office. I was reading the day’s mail before unfolding my couch and crashing. I came across an interview from the Colorado Physicians Health Program, with the widow of a resident who had overdosed on Fentanyl. Before I had finished reading it, I was in tears. It occurred to me that this tragedy could be put to some creative use. Along with a colleague at the University of California, San Francisco, Ted Eger, and a professional film maker friend, Dirk Wales, we decided that if this resident’s widow was willing, we would make a video of her recounting her loss—extremely powerful footage talking about the experience and the denial on both her and her husband’s parts, and then, of course, the tragic ending. In time the video, titled *Wearing Masks,* was distributed to all the hundred-plus anaesthesia-training programmes around the country. It is shared with all new residents and their partners in the hope that help will be sought by one or the other before it’s too late.

## Q. Your involvement in altitude-related research led to you co-editing the very well-known *High Altitude: An Exploration of Human Adaptation* with Robert Schoene. How did this come about?

That all started in 1974 when I was in Kashmir at a symposium preceding the World Physiology Congress being held in New Delhi. A very dynamic colleague, Claude Lenfant, then head of the National Heart Lung Blood Institute of the NIH, was producing a series of books called *Lung Biology in Health and Disease,* and he wanted one on the control of breathing. Well, I didn’t really want to do it, for the idea of editing a book didn’t turn me on, but Claude kept putting the screws on me and eventually I accepted and put together a two-volume set on Regulation of Breathing. One lesson I learned from that experience was that I would never do it again. Badgering busy people to submit their chapters on time was like pulling teeth. In the early ‘90s Claude asked if I could suggest someone who might want to do a book on acclimatisation to high altitude. I guess I must have forgotten what I’d learned, for I said I might be interested. I decided I wanted a book that people not in the direct area could understand and read with some pleasure. So a young colleague, Brownie Schoene, and I pulled together the big guns in the field and had a lot of fun doing together *High Altitude: an Exploration of Human Adaptation*…though there were still a few sluggish contributors.

## Q. You’re still as keen on the outdoor life as always. What fills your time now, apart from your regular climbs and walks?

The Altitude Research Centre (ARC) at the University of Colorado Medical School in Denver is one of the meaningful things in my life now. It’s evolving in interesting ways and I think I’ve been able to be helpful with that, and hopefully will continue to be. Colorado is the highest state in the US, and we’ve a growing population living as I do, at 7000–10,000 feet, who are getting older. Little is known about how illnesses of ageing interact with altitude—for better or worse. So there’s obviously tremendous potential for enquiry, and we’re just really beginning to get that off the ground with some of these communities up in the mountains.

One evolution of my life as a doc is to help older people find good medical care for serious illnesses. From my past academic life, I’ve a network all over the United States: in essence, where I try and find good care for people. I sort-of serve a triage function helping people to find help. My mantra, as a once-physician, in this phase of my life is ‘second opinion’, because when people get serious illnesses I am very aware of just how finite each of our individual lives is. Even if you’ve got a good doc, if you’ve got a bad situation, it’s awfully good to get a second skilled doc evaluating what’s going on before you make serious commitments to this or that therapy.

I have also been thinking a lot about patient care, perhaps because I am more recipient than donor at this stage in my life. In some ways the medical system here has become very efficient. You go to a specialist’s office and are greeted by very well trained, personable personnel, but it’s like a Toyota factory: efficient and programmed, but something is missing. It struck me that the weak point is the doc at the back end of the assembly line—usually pleasant but seemingly always pressed for time. A doctor–patient relationship is often hard to come by and may be an unrealistic expectation. But patients are people, each with his or her own uniqueness of dreams, aspirations, and needs. It takes a little time and commitment, and some risk-taking, to be both caregiver and carer to one’s patients, coming down to genuine caring and empathy. During my years as an anaesthesiologist, it took me a while to develop the confidence and wisdom to appreciate the preciousness of just being a caring person and being able to give of yourself in that way. Eventually I figured I was worth at least 10 mg of Diazepam. I could walk up to a patient on a gurney before surgery, someone who was clearly petrified and trying to be brave, put my hands on them, and just watch them relax. I tried by example to be a role model for the residents I worked with, and I tended to think that teaching them about compassion was just as important as anything else I could teach them, even though it wasn’t rocket science or profound physiology. Patients are people, with their dreams, their fears, and long lives lived.

Also as an ex-doc, I have also been involved in the dyings of friends in recent years. You come to realise at this stage in life, as many have observed before me, that the transition of your friends from a state of vitality to one of memory is an inevitable part of the game so long as you are still around to be an observer. I look at these individuals who have been cherished friends, many from my mountaineering community, and there seems to be one common denominator to their mode of exiting: these are people who have lived their life on the edge. They are not risk averse. They have a sense of the preciousness of life, and they also are aware that, as Gil Roberts, our Everest doc, said, ‘Nobody gets out of this life alive’. When the time comes, they take it in their stride and often try to give meaning with their exiting to the lives of those left behind, a priceless gift. With his dying, Barry Corbet, one of our West Ridge team, left me realising that for him dying was still part of life, its final chapter. It deserves to be done in style, just as he lived the rest of life. Death is the endpoint of dying.

And finally there’s a lot going on in the mountaineering part of my life—work that relates to the political and safety side of it. One bit relates to Everest and its overcrowding. In addition to the inherent risks that the mountain brings in terms of weather, falling objects, and altitude, there’s the risk brought about by too many people being in the same place at the same time. It makes for bigger targets in the bowling alley when you can only move at the rate of the slowest person. I don’t question why people climb the mountain, but the consequence of it has become that there are too many, adding man-made risks to the ample supply already inherent in climbing big mountains. So, with others, I have tried to catalyse some thinking on how to effect change on the mountain and within the Sherpa community that lives beneath it.

If I look at my life and try to characterise my modus in one word, it would be ‘catalyst’. I lack the talent to do a lot of the things that need to be done, but I seem able to help other people pick up the reins and really do some magnificent things far beyond my own capacity. That’s occurred in a whole lot of different arenas: mountaineering, academia, and in other areas too.

## Q. But you also have the capacity to be a musician!

Well, not really. It’s been a big challenge to learn how to play the piano starting too late in life. I actually began in my mid-forties for about 3 years, but then I became department Chair and our daughter was born, so I just couldn’t find the time to practise. When we moved to Colorado, I was told in no uncertain terms, ‘If you’re taking that piano with you, you’d better damn well play it.’ So I do, and I’ve been taking lessons. It’s slow, plenty challenging, and I enjoy it. I’ll never be good, but it doesn’t matter. Just starting a new piece and eventually getting to the place where I can revel in the beauty of the music, even though it’s never perfect, is music for the soul. I perform for me, not for anyone else; an act of kindness on my part.

## Q. Finally, a few generic questions if I may? A number of people have influenced your career. Do any particular people stand out as key influences?

Mentors are priceless and I’ve been gifted many. Albert Roos, a Dutchman who escaped to the US just before the Germans moved into Holland in WWII, was perhaps my most precious mentor and friend to his end. John Severinghaus has been guide, mentor, and friend for many years. Another was Julius Comroe. He was one of those bigger-than-life figures to our generation: thoughtful, critical, articulate, and a very caring human being. I gave a history talk at Colombia University Medical School several decades ago. I had been thinking about heroes and mentors, and I decided I would talk about Comroe with this anaesthetic audience. So I asked, ‘Who’s heard of Julius Comroe?’ And about four hands in the audience went up, and they all had grey hair! So, if you’re not a Mozart, one’s fame and mortality are evanescent. Julius, and Robert Dripps were amongst a handful of that generation of very inspiring, wise, thoughtful, and nurturing human beings. Then there was Henrik Bendixen, a Norwegian-turned-Dane, who was one of the pioneers of critical care medicine back at the Massachusetts General Hospital; Henrik helped expand the horizons of my thinking and reading into domains I would have never ventured into, and probably gets the blame, along with my boss, John Bonica, for seeding whatever leadership roles I played in medicine.

